# Designing a novel multi-epitope vaccine to evoke a robust immune response against pathogenic multidrug-resistant *Enterococcus faecium* bacterium

**DOI:** 10.1186/s13099-022-00495-z

**Published:** 2022-05-27

**Authors:** Jyotirmayee Dey, Soumya Ranjan Mahapatra, T. Kiran Raj, Taranjeet Kaur, Parul Jain, Arushi Tiwari, Shubhransu Patro, Namrata Misra, Mrutyunjay Suar

**Affiliations:** 1 School of Biotechnology, Kalinga Institute of Industrial Technology (KIIT), Deemed to Be University, Campus-11, Bhubaneswar, Odisha 751024 India; 2 Department of Biotechnology & Bioinformatics, School of Life Sciences, JSS Academy of Higher Education & Research, Mysuru, India; 3 Biotechnology Industry Research Assistance Council (BIRAC), New Delhi, India; 4 Kalinga Institute of Medical Sciences, KIIT Deemed to Be University, Bhubaneswar, India; 5 KIIT-Technology Business Incubator (KIIT-TBI), Kalinga Institute of Industrial Technology (KIIT), Deemed to Be University, 751024, Bhubaneswar, Odisha India

**Keywords:** *Enterococcus faecium*, Penicillin-binding protein, Vaccine, Epitope, Immunoinformatics

## Abstract

*Enterococcus faecium* is an emerging ESKAPE bacterium that is capable of causing severe public health complications in humans. There are currently no licensed treatments or vaccinations to combat the deadly pathogen. We aimed to design a potent and novel prophylactic chimeric vaccine against *E. faecium* through an immunoinformatics approach The antigenic Penicillin-binding protein 5 (PBP 5) protein was selected to identify B and T cell epitopes, followed by conservancy analysis, population coverage, physiochemical assessment, secondary and tertiary structural analysis. Using various immunoinformatics methods and tools, two linear B-cell epitopes, five CTL epitopes, and two HTL epitopes were finally selected for vaccine development. The constructed vaccine was determined to be highly immunogenic, cytokine-producing, antigenic, non-toxic, non-allergenic, and stable, as well as potentially effective against *E. faecium*. In addition, disulfide engineering, codon adaptation, and in silico cloning, were used to improve stability and expression efficiency in the host *E. coli*. Molecular docking and molecular dynamics simulations indicated that the structure of the vaccine is stable and has a high affinity for the TLR4 receptor. The immune simulation results revealed that both B and T cells had an increased response to the vaccination component. Conclusively, the in-depth in silico analysis suggests, the proposed vaccine to elicit a robust immune response against *E. faecium* infection and hence a promising target for further experimental trials.

**Supplementary Information:**

The online version contains supplementary material available at 10.1186/s13099-022-00495-z.

## Introduction

*Enterococcus faecium* *is* among the most frequent causes of healthcare-associated infections with underlying co-morbidities such as UTIs, intra-abdominal infection, prostatitis, organ transplantation, diabetes, endocarditis [1]. *E. faecium* is also reported as the second most prevalent organism involved in bloodstream infections and rank fourth in terms of surgical site infections in the US and European hospitals [2, 3]. Cassini et al. reported 16,146 instances of vancomycin-resistant enterococcal infections and 1081 related fatalities in Europe in 2015 [4]. According to Ferede et al. the prevalence rate of *E. faecium* in India was found to be 2.3% [5]. On average, 2.5 million people become infected with antibiotic-resistant *E. faecium,* and more than 25,000 people die each year across the world. The annual incidence of *E. faecium* is 1.6 per 100,000 population [6]. The World Health Organization issued a list of 12 antibiotic-resistant pathogens in 2017 posing the greatest threat to human health, with *E. faecium* designated as a high priority “ESKAPE pathogen” for the discovery of novel therapies [7].

Several putative virulence factors have been reported in *E. faecium* such as Enterococcal Surface Protein, aggregation substance, pili, MSCRAMMs (microbial surface components recognizing adhesive matrix molecules), cell wall, capsular polysaccharides, glycolipids, gelatinase, and fsr two-component system. These proteins are mainly involved in adherence to extracellular structures and biofilm formation, important processes in initiating colonization of and infection in the host. In Gram-positive bacteria *E. faecium*, the cell wall is composed of a peptidoglycan macromolecule that protects bacteria against environmental conditions and serves as an anchor for the attachment of capsular polysaccharides, teichoic acids, and proteins that are covalently or non-covalently attached to peptidoglycan [8]. Bacterial peptidoglycan polymerization is performed by multi enzymatic complexes that include high-molecular-weight class A and B penicillin-binding proteins (PBPs) [9]. PBPs are transpeptidases, carboxypeptidases, and endopeptidases that synthesize new and remodel existing peptidoglycan, the stress-bearing component of the bacterial cell wall [10]. They help to create the morphology of the peptidoglycan exoskeleton together with cytoskeleton proteins that regulate septum formation and cell shape. Class A PBPs combine the two activities essential for peptidoglycan polymerization, glycosyltransferase, and d,d-transpeptidase, in a single polypeptide chain. Class B PBPs are monofunctional d,d-transpeptidases that must cooperate with a glycosyltransferase to synthesize peptidoglycan. In *E. faecium*, resistance to β-lactam antibiotics is conferred by a low-affinity class B PBP, PBP 5. PBP 5 is the major carboxypeptidases that play a key role in the control of cell diameter, proper spore cortex synthesis, and correct septum formation [9, 11]. Due to its capacity to evolve toward high-level resistance in *E. faecium*, PBP 5 is widely reported as an attractive target for vaccine development [11].

Glycopeptides, penicillin, aminoglycosides, vancomycin macrolides, oxazolidinones, clindamycin, cephalosporins, trimethoprim/sulfamethoxazole, and beta-lactams are the major antibiotics available for the treatment of infections caused due to *E. faecium*. However, due to increased resistance to β-lactam, glycopeptide vancomycin was the antibiotic employed to treat these infections. In the last decade, the number of vancomycin-resistant enterococci (VRE) has increased in health care facilities globally. Infection caused by VRE leads to poor outcomes, and it remains a challenge. Quinupristin/dalfopristin, daptomycin, tigecycline, and linezolid have entered the clinical practice as alternative antibiotic agents to fight VRE infections; however, *E. faecium* is resistant to these agents has also emerged and are reported recently.

Combating multidrug-resistant *E. faecium* infections with newer medicines necessitates long-term treatments during which resistance may emerge, leaving physicians with limited treatment alternatives [12]. Several world wide efforts have been undertaken to fight agsinst the infection. For instance, Romero-Saavedra et al. identified six enterococcal proteins that could serve as potential vaccine candidates against enterococcal infections by the implementation of transcriptomic and proteomic approaches [13, 14]. In both studies, rabbits were immunized with the recombinant proteins, and the resulting sera were evaluated. Both results indicate the potential use of these proteins as vaccine candidates with a broad cross-reactivity and serotype-independent coverage against enterococcal infections. A few studies [14, 15] have also reported the efficacy of glycoconjugate vaccines as they induced opsonic antibodies against Enterococcus species experimentally. Despite these advances, complete protection against this infection is yet to be achieved. As a consequence, there is an urgent need to develop alternate vaccine-based strategies to prevent infections. Reverse vaccinology is a method that has revolutionized vaccine development in the past [16]. This allows the development of the novel vaccine antigens from the genome sequence information without the requirement to isolate and culture the pathogen [17].

This research article was developed with the objective of designing a potent in silico multi-peptide vaccine for *E. faecium* infection. The initial step of the vaccine development process was to identify epitopes that can be used as immunogens. Predicted epitopes with high antigenicity, non-allergenicity, nontoxicity, and positive immunogenicity score were combined with the linkers. An adjuvant was added to the N-terminal of the antigenic epitope to make the multiepitope vaccine more immunogenic. Different bioinformatics tools were used to investigate the physicochemical, structural, and immunological aspects of the final vaccine construct. Furthermore, the chimeric vaccine sequence has been codon-optimized for expression in *E. coli*.

## Methodology

**Fig. 1:**
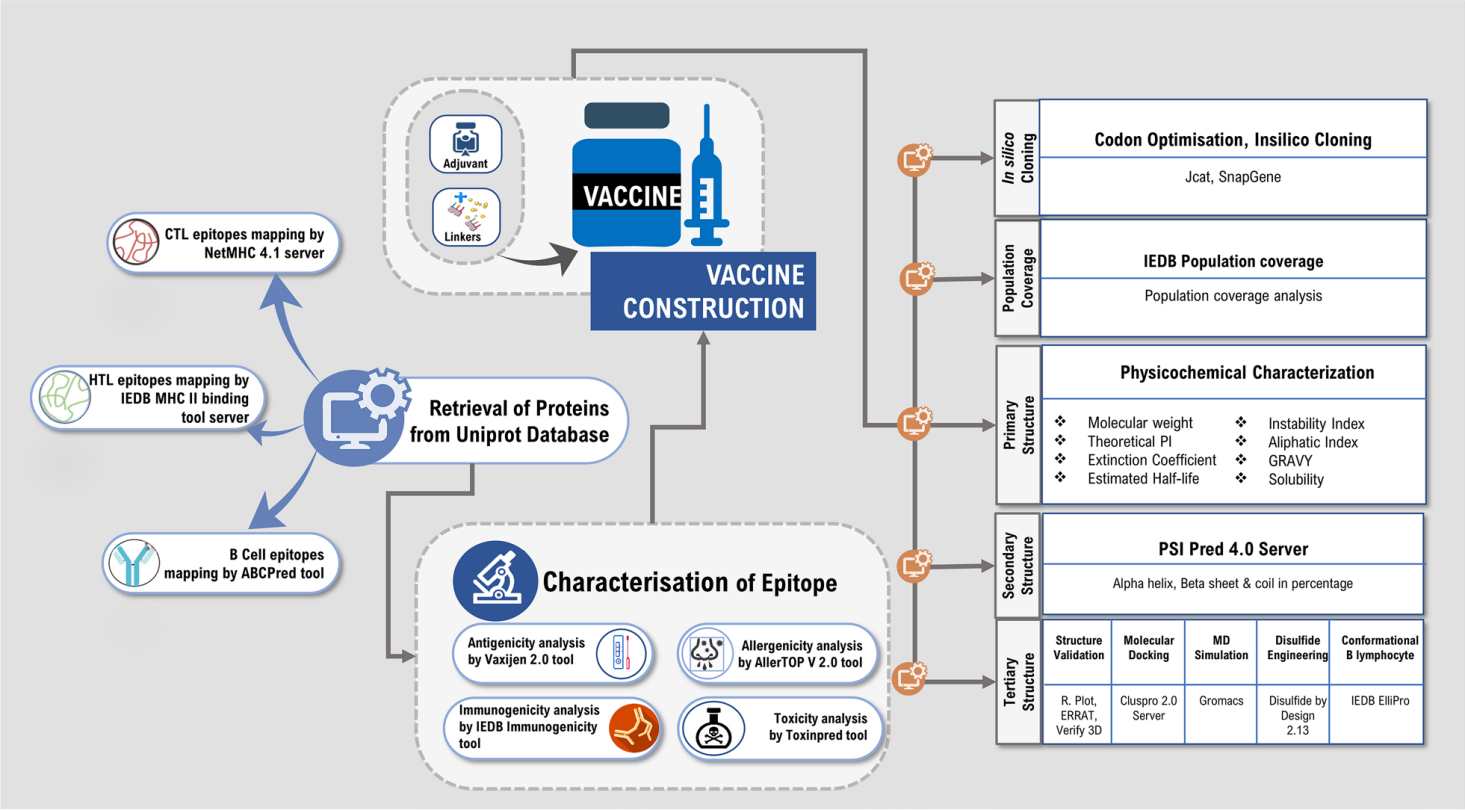
The schematic representation of the immunoinformatics guided design of the multi-epitope vaccine construct against *Enterococcus faecium*

The pictorial representation of the workflow is depicted in Fig. 1.

### Retrieval of protein sequence

The amino acid sequence of the major secreted protein, Penicillin-binding protein 5 (PBP 5) (accession id: G5CKR9) of *Enterococcus faecium* was obtained from the UniProt database (https://www.uniprot.org/). The accession Id of the protein was derived from the manual search in the search bar of the UniProt database.

### Linear B lymphocyte (LBL) epitope prediction

The ABCpred server (http://crdd.osdd.net/raghava/abcpred/ABC_submission.html) was used to predict linear B cell epitopes. FASTA sequence with a window length of 10 was given as input and the program provided 10mer epitopes with a default specificity of 75% for B-cell receptors. The epitope with the best score was chosen for the future development of a multi-epitope vaccine [18].

### Cytotoxic T lymphocyte (CTL) epitope prediction

Prediction of CTL epitopes of the target protein was rendered using NetMHCpan4.1 (http://www.cbs.dtu.dk/services/NetMHCpan-4.1/) server. The threshold for epitope identification for a strong binder was set at 0.5% while at 2% for a weak binder. The server predicts epitopes for 12 HLA class I supertypes. The epitopes from each supertype were predicted. ‘Sort by Prediction score’ was selected. Prior to prediction, all the epitope lengths were set as 9mers, conserved epitopes that bind to many HLA alleles [19].

### Helper T lymphocyte (HTL) epitope prediction

For predicting (15mer) HTL, the selected protein was given as input in the IEDB (Immune Epitope Database) MHC II binding tool. The HTL epitopes were predicted using the whole HLA (Human leukocyte antigen) reference set. It covers all HLA class II alleles including HLA-DR, HLA-DQ, and HLA-DP. The suggested IEDB parameters were utilized to make the prediction, which included a complete set of 27 human alleles that covered 99 %of the population. The window length was kept at 15 and the peptides were sorted according to the adjusted rank. HTL epitopes were chosen based on their IC50 (Inhibition concentration 50) values, the epitopes with low IC50 values (IC50 value represents the minimal concentration of a drug that is required for 50% inhibition in vitro) displaying strong affinity [20].

### Antigenicity, allergenicity, and toxicity assessment

The antigenicity was predicted using the Vaxijen v2.0 server (http://www.ddg-pharmfac.net/vaxijen/VaxiJen/VaxiJen.html). This software requires epitope sequence to certify antigen classification uniquely based on the physicochemical properties. In contrast to bacterial species, the default settings (threshold = 0.4, ACC output) were utilized to predict the antigenicity of the epitopes sequence [21].

AllerTOP v. 2.0 (https://www.ddg-pharmfac.net/AllerTOP/feedback.py), a bioinformatics tool for allergenicity prediction, was used to estimate if epitopes extracted from bacterial protein sequences would cause an allergic reaction in humans. To calculate the allergenicity of the sequence, all of the epitopes in the plain text were used as input [22].

ToxinPred (https://webs.iiitd.edu.in/raghava/toxinpred/multi/submit.php) was used to forecast the toxicity of specified epitopes. All peptides were entered in FASTA format, and all other settings were left at their default values [23].

Another tool from the IEDB Analysis Resource, Class I Immunogenicity (http://tools.iedb.org/immunogenicity/), was used to estimate the degree of immunogenicity of the chosen peptides. The epitopes were provided as input, with the rest of the settings set to default [24].

### Population coverage analysis

The population of anticipated epitopes was determined using the IEDB population coverage tool (http://tools.iedb.org/population/). To perform predictions, the epitopes and alleles were given as inputs. The IEDB recommended method is the default setting and usually is consensus, a combination of three different methods (ANN, SMM, and Combinatorial Library). For humans, the most frequent alleles are available for selection by default [25].

### Construction of multi-epitope vaccine

Because of their small size, epitopes are often non-immunogenic when used alone as a vaccine [26]. To activate the innate and adaptive immune systems, they need a carrier with potent immuno-stimulatory adjuvants. Linkers play a critical role in replicating the vaccine construct's ability to serve as an independent immunogen and produce higher antibody concentrations than a single immunogen [27]. To boost the stability and antigenicity of the final vaccine, selected LBL, CTL, and HTL epitopes were connected with linkers such as KK, AAY, and GPGPG, respectively. LBL epitopes were joined with ‘KK’ linkers, CTL epitopes were joined with ‘AAY’ linkers, and HTL epitopes were joined with ‘GPGPG’ linkers. Adjuvant was attached with the help of EAAAK linker to the N terminal end of the vaccine.

### Physiochemical properties of the constructed vaccine

The ProtParam server (https://web.expasy.org/protparam/) was used to determine the physical and chemical characteristics of the final vaccine, including amino acid composition, theoretical pI, instability index, in-vitro and in-vivo half-life, aliphatic index, molecular weight, and grand average of hydropathicity (GRAVY).

### Secondary structure prediction

PSIPRED (http://bioinf.cs.ucl.ac.uk/) was used to estimate the secondary structure of the vaccine's main amino acid sequence. ‘PSIPRED 4.0 (Predict secondary structure)’ option was chosen from the ‘popular analyses’ section [28]. The complete sequence was analyzed by default.

### Predicting tertiary structure and validation

The predicted subunit vaccine candidate's 3D structure was retrieved using the Robetta internet server (https://robetta.bakerlab.org/). For structure prediction, the target name and vaccine sequence was submitted to the server. The tertiary structure was provided through the web interface. The visualization of the structure was done using the pymol visualization software. After that, the validation of the protein structure was performed through PROCHECK (https://servicesn.mbi.ucla.edu/SAVES/), ERRAT (https://servicesn.mbi.ucla.edu/SAVES/), and Verify 3D (https://servicesn.mbi.ucla.edu/SAVES/) online servers. The structure was submitted to these servers in PDB (Protein Data Bank) format.

### Disulphide engineering of the final vaccine construct

To boost stability, the vaccine structure was submitted to the Disulphide by Design (DbD) 2.12 tool (http://cptweb.cpt.wayne.edu/DbD2/). Potential residue pairings were chosen based on a set of parameters (87 to + 97 chi3 value and < 2.2 energy value) and mutated with a cysteine residue [29].

### Discontinuous B-cell epitope prediction

To anticipate the discontinuous B-cell epitopes in the final vaccine structure, the ElliPro server (http://tools.iedb.org/ellipro/) was employed [30]. The PDB structure was given as input. The threshold values were set at default. The minimum residue score (protrusion index) was between 0.5 and 1.0 and the maximum distance was in the range 4–8 Å. It assigns an ellipsoid score to each residue, which is characterized as a PI (Protrusion Index) value based on the 3D structure of the vaccine.

### Molecular docking

The tertiary structure of the muti-epitope vaccine was examined as a ligand molecule, and TLR-4 (PDB ID: 5IJB) was chosen as the receptor. Docking was performed by the Autodock 4.0 program using the empirical free energy function and the Lamarckian Genetic algorithm [31]. Partial charges were added to the ligand, and the non-polar hydrogens were linked. The grid map was created with Autogrid, with a grid box diameter of 90*90*90 and a spacing of 0.35 angstrom. Based on the reactive distances and binding energies, the optimum conformation of the interaction was determined after docking.

### Molecular dynamics simulation

Molecular dynamics simulations were performed using the GROMACS-2019.4 version [32, 33] using the Gromos force field and SPC-2 water model. Energy minimization was used to remove high energy configurations (bonded terms distant from equilibrium or steric conflicts). The system then proceeds to equilibrate the atoms of molecules with NVT for 500 ps at a temperature of 300 K. Similarly, the NPT equilibrium operates at 1 bar pressure and 300 K. Following the equilibration procedure, a 100 ns MD simulation of the receptor (TLR-4) and ligand (final vaccine design) complex was performed. The trajectory file obtained by the MD simulation was examined using the GROMACS commands gmx rms, gmx rmsf, gmx gyrate, and gmx hbond to obtain the root-mean-square deviation (RMSD), root mean square fluctuation (RMSF), radius of gyration, and hydrogen bond interaction, respectively.

### *Codon adaptation and *in silico* cloning of the vaccine*

The greater expression rates might be due to a distinct codon adaptation technique that was adapted to *E. coli* K12, the most sequenced prokaryotic organism. In the instance of the host organism *E. coli* K12, the approach was utilized to increase the expression of the main sequence of the subunit vaccination protein, which was subsequently sent to the JAVA Codon Adaptation Tool. Prokaryote ribosome binding site, rho independent transcription termination, and restriction enzyme cleavage site were all avoided. Using the restriction cloning module of the SnapGene program, the altered nucleotide sequences of the developed multi-subunit vaccine were further cloned into the *E. coli* pET-28a ( +) vector between restriction sites (GSL Biotech, available at snapgene.com).

### Immune simulation

Immune simulations were run on the CImmSim server (http://150.146.2.1/C-IMMSIM/index.php) to verify the expected MEV's immune response. The MEV's capacity to activate immune system cells such as NK cells, HTL, B-cells, CTL, immunoglobulins, cytokines, and dendritic cells was investigated. A four-week interval between two vaccination doses is advised in clinical practice. Immune simulation was done using a technique that was similar to this system. In a nutshell, every 4 weeks, three shots were given. The total number of simulation steps was 1050.

## Results

### Criteria for epitope prediction

Toxic and allergic epitopes should be avoided since they can endanger the goal of vaccine development. Antigenicity, allergenicity, toxicity, and immunogenicity of anticipated epitopes were assessed using Vaxigen v2.0, AllerTOP v2.0, Toxinpred, and the IEDB class I immunogenicity tool, respectively. For further investigation, epitopes with positive immunogenicity scores, high antigenicity, non-allergenicity, and nontoxic nature were chosen.

### Prediction of linear B-cell epitopes

The ABCpred server was used to examine the protein's amino acid sequences for the presence of linear epitopes. A total of 30 epitopes of length 10 mer were predicted as mentioned in Additional file 1: Table S1. Only two epitopes (DADGVEKKVL and DIKLTIDAKA) were chosen for the final vaccine construct based on the above-mentioned criteria (Table 1).

**Table 1:** Final selected B-cell epitopes from *Enterococcus faecium* penicillin binding protein and their corresponding immunogenic properties

Uniprot_ID	B-cell epitope	Position	Score	Antigencity score	Toxicity	Hydrophobicity	Hydropathicity	Hydrophilicity	Charge	Mol wt
G5CKR9	DADGVEKKVL	323	0.77	1.905	Non-toxin	− 0.22	− 0.47	0.97	− 1	1073.35
	DIKLTIDAKA	342	0.71	1.8105	Non-toxin	− 0.13	0.09	0.52	0	1087.42

### Prediction of cytotoxic T lymphocytes epitope

MHC Class I binding peptides were predicted using the NetMHCpan 4.1 server (Additional file 2: Table S2). The top five peptides from having high antigenicity and positive immunogenicity score were shortlisted for the final vaccine construct and are shown in Table 2.

**Table 2:** Predicted CTL epitopes from *Enterococcus faecium* penicillin binding protein to design multi-epitope vaccine construct with their corresponding MHC Class I alleles and their immunogenic properties

Uniprot_ID	CTL epitope	Alleles	Position	Score	Antigencity score	Immunogenicity	Toxicity	Hydrophobicity	Hydropathicity	Hydrophilicity	Charge	Mol wt
G5CKR9	KLGDGGEKT	HLA-A*01:01, HLA-A*02:01, HLA-A*03:01, HLA-A*24:02, HLA-A*26:01, HLA-B*07:02, HLA-B*08:01, HLA-B*27:05, HLA-B*39:01, HLA-B*40:01, HLA-B*58:01, HLA-B*15:01	199	3.466	2.5192	0.05672	Non-toxin	− 0.3	− 1.43	1.09	0	904.12
	KLIADKETK	HLA-A*03:01, HLA-A*26:01, HLA-B*07:02, HLA-B*08:01, HLA-B*27:05, HLA-B*39:01, HLA-B*40:01, HLA-A*01:01, HLA-A*02:01, HLA-A*24:02, HLA-B*58:01, HLA-B*15:01	567	0.395	0.4315	0.00835	Non-toxin	− 0.37	− 1.03	1.17	1	1045.37
	TTINWQPNL	HLA-A*24:02, HLA-B*07:02, HLA-A*01:01, HLA-A*02:01, HLA-B*08:01, HLA-A*03:01, HLA-A*26:01, HLA-B*27:05, HLA-B*39:01, HLA-B*40:01, HLA-B*58:01, HLA-B*15:01	145	3.248	1.4839	0.13021	Non-toxin	− 0.09	− 0.68	− 0.8	0	1086.35
	EVDGRYYPL	HLA-B*58:01, HLA-A*01:01, HLA-A*02:01, HLA-A*03:01, HLA-A*24:02, HLA-A*26:01, HLA-B*07:02, HLA-B*08:01, HLA-B*27:05, HLA-B*39:01, HLA-B*40:01, HLA-B*15:01	259	0.794	1.6871	0.07862	Non-toxin	− 0.21	− 0.9	0.12	− 1	1111.34
	LRGTTGGKL	HLA-B*27:05, HLA-A*24:02, HLA-A*26:01, HLA-B*07:02, HLA-B*58:01, HLA-A*01:01, HLA-A*02:01, HLA-A*03:01, HLA-B*08:01, HLA-B*39:01, HLA-B*40:01, HLA-B*15:01	311	2.321	1.9864	2.321	Non-toxin	− 0.19	− 0.38	0.18	2	902.2

### Prediction of helper T lymphocytes epitope

Peptides that bind to MHC class II molecules were predicted using the IEDB server's MHC class II binding peptide prediction tool. The IEDB-recommended approach was employed for MHC class II binding, and a reference set of 27 alleles was used. As shown in Additional file 3: Table S3, a total of 10 HTL epitopes and their matching HLA class II binding alleles were predicted for the protein sequence. Based on the above criteria, the top two HTL epitopes (DKFIFGEDLDLPISM and DSLGGKAGSTVATTP) were selected for the final vaccine construction (Table 3).

**Table 3:** Predicted HTL epitopes from *Enterococcus faecium* penicillin binding protein to design multi-epitope vaccine construct with their corresponding MHC Class II alleles and their immunogenic properties

Uniprot_ID	MHC II epitope	Alleles	Pos	IC50 value	Percentile_Rank	Antigencity Score	Toxicity	Hydrophobicity	Hydropathicity	Hydrophilicity	Charge	Mol wt
G5CKR9	DKFIFGEDLDLPISM	HLA-DRB1*01:01, HLA-DPA1*01:03, HLA-DPB1*02:01, HLA-DPB1*01:01, HLA-DRB1*09:01,HLA-DRB3*02:02, HLA-DRB1*13:02, HLA-DRB1*11:01, HLA-DRB1*04:01, HLA-DRB1*12:01, HLA-DPA1*03:01, HLA-DPB1*04:02, HLA-DRB1*04:05, HLA-DRB1*15:01, HLA-DQA1*01:01, HLA-DQB1*05:01, HLA-DRB1*08:02, HLA-DPA1*02:01, HLA-DPB1*14:01, HLA-DPB1*04:01, HLA-DQA1*05:01, HLA-DQB1*03:01, HLA-DQA1*04:01, HLA-DQB1*04:02, HLA-DPA1*02:01, HLA-DPA1*02:01, HLA-DPB1*05:01, HLA-DPA1*01:03, HLA-DQA1*05:01, HLA-DQB1*02:01, HLA-DQA1*03:01, HLA-DQB1*03:02, HLA-DQA1*01:02, HLA-DQB1*06:02, HLA-DRB3*01:01, HLA-DRB5*01:01, HLA-DRB1*07:01, HLA-DRB4*01:01, HLA-DRB1*03:01	502–516	19	0.87	0.8406	Non-toxin	0	0.23	0.12	− 3	1740.22
	DSLGGKAGSTVATTP	HLA-DRB5*01:01, HLA-DRB1*15:01, HLA-DRB3*01:01, HLA-DPA1*03:01, HLA-DPB1*04:02, HLA-DPA1*01:03, HLA-DPB1*02:01,HLA-DRB1*01:01, HLA-DRB1*09:01,HLA-DRB3*02:02, HLA-DRB1*13:02, HLA-DRB1*11:01, HLA-DRB1*04:01, HLA-DRB1*12:01,HLA-DRB1*04:05, HLA-DQA1*01:01, HLA-DQB1*05:01, HLA-DRB1*08:02, HLA-DPA1*02:01, HLA-DPB1*14:01, HLA-DPA1*01:03, HLA-DPB1*04:01, HLA-DQA1*05:01, HLA-DQB1*03:01, HLA-DQA1*04:01, HLA-DQB1*04:02, HLA-DPA1*02:01, HLA-DPB1*01:01, HLA-DPA1*02:01, HLA-DPB1*05:01,HLA-DQA1*05:01, HLA-DQB1*02:01, HLA-DQA1*03:01, HLA-DQB1*03:02, HLA-DQA1*01:02, HLA-DQB1*06:02, HLA-DRB1*07:01, HLA-DRB4*01:01, HLA-DRB1*03:01	357–371	12	1.3	1.9712	Non-toxin	− 0.06	− 0.15	0.07	0	1361.68

### Population coverage analysis of the selected epitopes

Individual and combinatory population coverage for the seven chosen T-cell epitopes (5 CTL and 2 HTL) with their binding HLA alleles was calculated, as shown in Additional file 4: Table S4. Global population coverage of 99.25% and 96.20% individuals will respond to the selected epitopes in India. West Africa had the highest population coverage of 99.93%.

### Construction of the multitope vaccine candidate

**Fig. 2:**
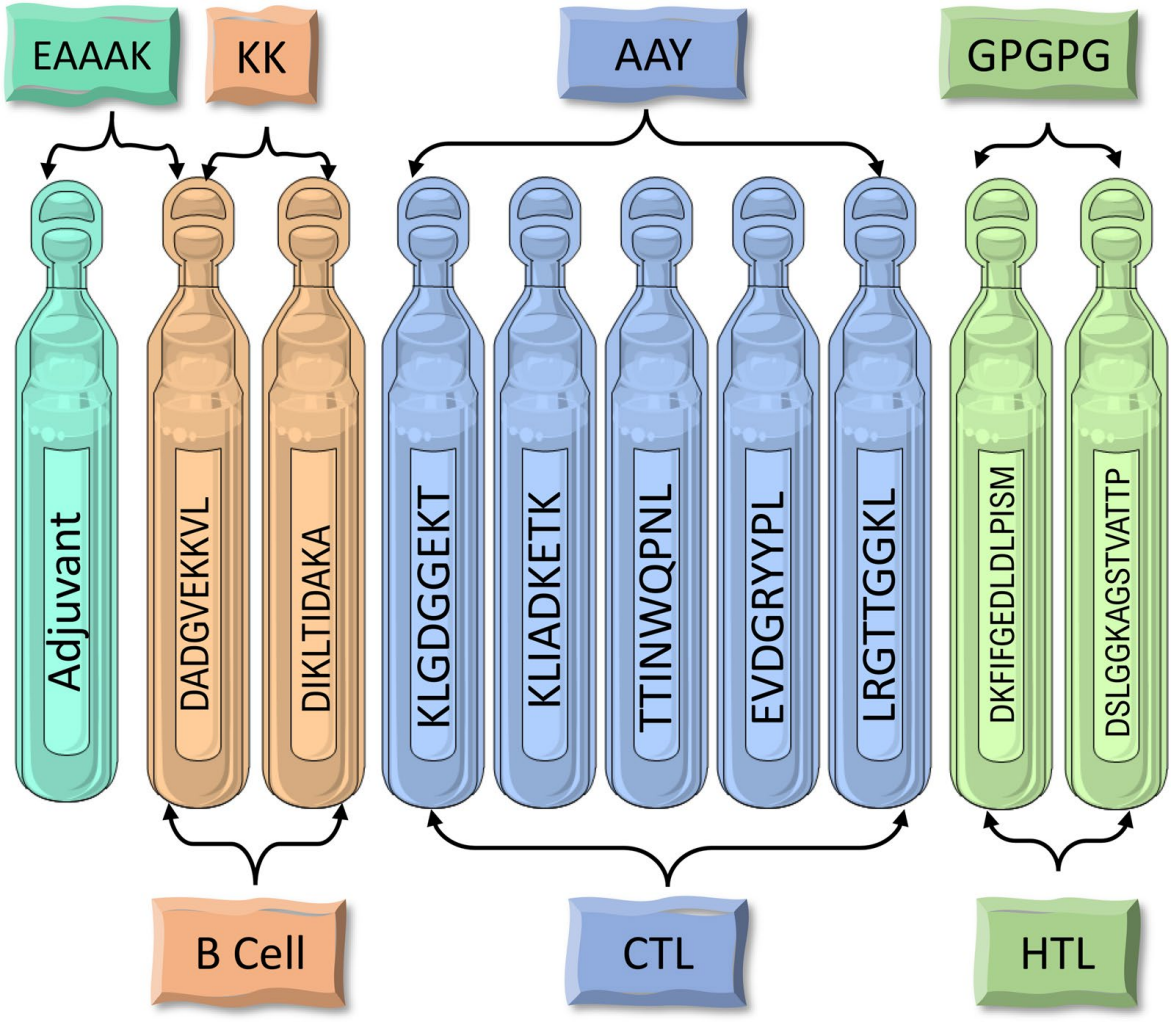
The structural arrangement of B and T-cell epitopes along with linkers and adjuvant for the final multi-epitope vaccine construct

Finally, a 172 amino acid long multi-peptide vaccine was created by connecting several epitopes such as LBL, CTL, HTL with suitable linkers at appropriate places. The final subunit vaccine was constructed using three linkers: KK, AAY, and GPGPG. The AAY and GPGPG linkers were introduced to the intra-epitope site to connect the CTL and HTL epitopes, respectively. Then, to boost the effectiveness of the immune response, human beta-defensins (accession id: P81534) of 67 amino acid length was added to the N-terminal end of the designed vaccine sequence as a molecular adjuvant. Figure 2 depicts the arrangement of adjuvant and epitopes with their connecting linkers.

### Physicochemical features, solubility, and secondary structure prediction

The aliphatic index, GRAVY, Theoretical PI, instability index, and molecular mass of the multi-epitope vaccine were calculated to be 73.90, −0.472, 9.38, 18.85, and 18,294.07 Da, respectively, based on the ProtParam server data. The assessment of the protein's molecular weight was the most important aspect throughout this phase [34, 35]. Proteins with a molecular weight of < 110 kDa are thought to be more suitable since they can be easily isolated and effectively put to vaccine development [36, 37]. Based on the study of physicochemical qualities, the vaccine design was anticipated to be stable, basic, hydrophilic, thermostable, and highly soluble following over-expression in *E. coli*. Furthermore, the vaccine's half-life was determined to be 30 h in human reticulocytes (in vitro), over 20 h in yeast (in vivo), and over 10 h in *E. coli* (in vivo). Additional file 5: Table S5 summarises the physicochemical parameter analysis results.

**Fig. 3:**
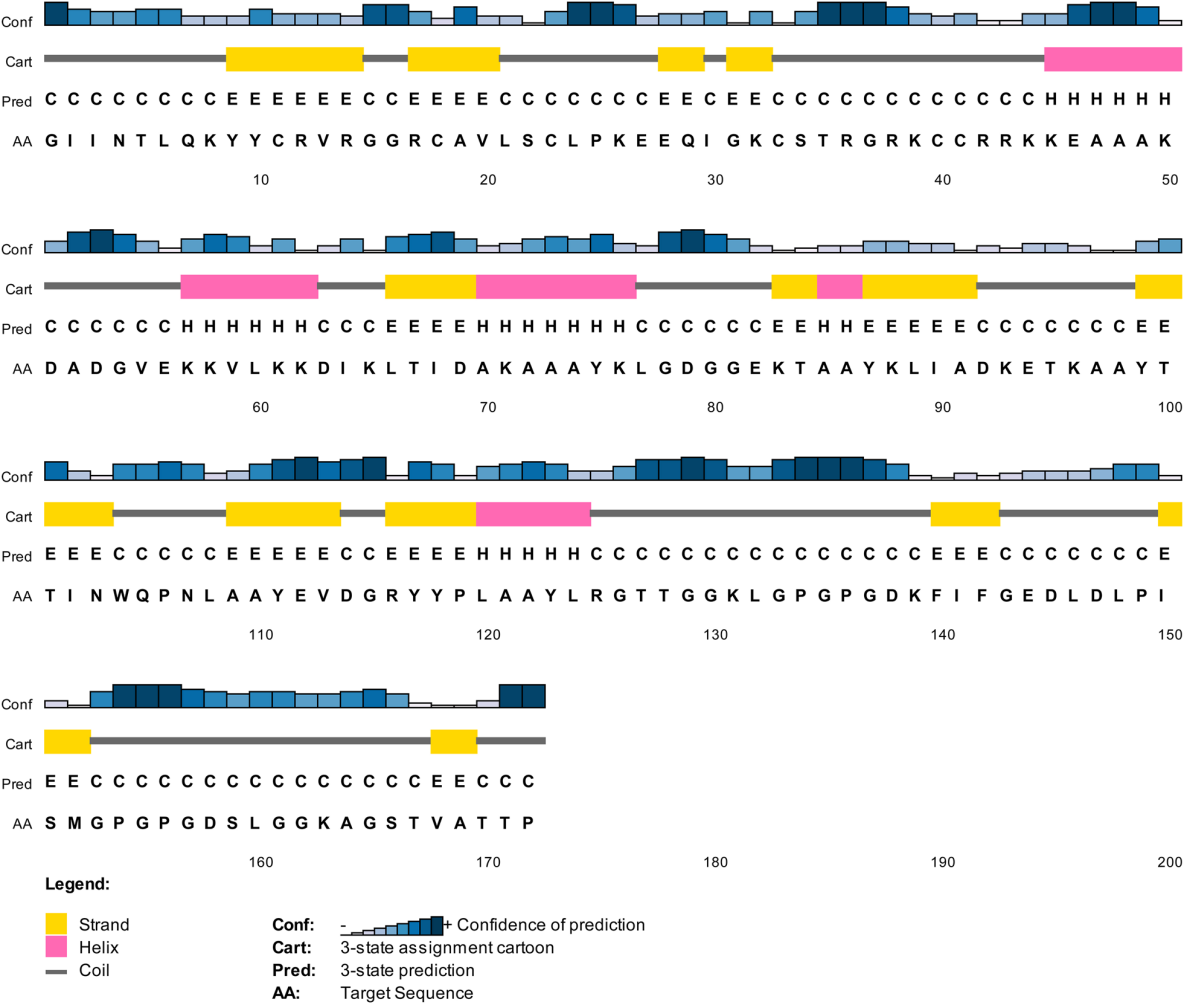
Secondary structure prediction of the final multi-epitope vaccine construct by using PSIPRED tool

According to the results of the PSIPRED server, random coils are formed by 99 amino acids, α-helix by 26 amino acids, and β-strands are formed only by 47 amino acids. The overall secondary structure prediction of the developed vaccine results concluded that 57.55% are random coils, 15.11% form α-helix and 27.32% are β-strands as represented in Fig. 3.

### Tertiary structure prediction and validation of the vaccine construct

**Fig. 4:**
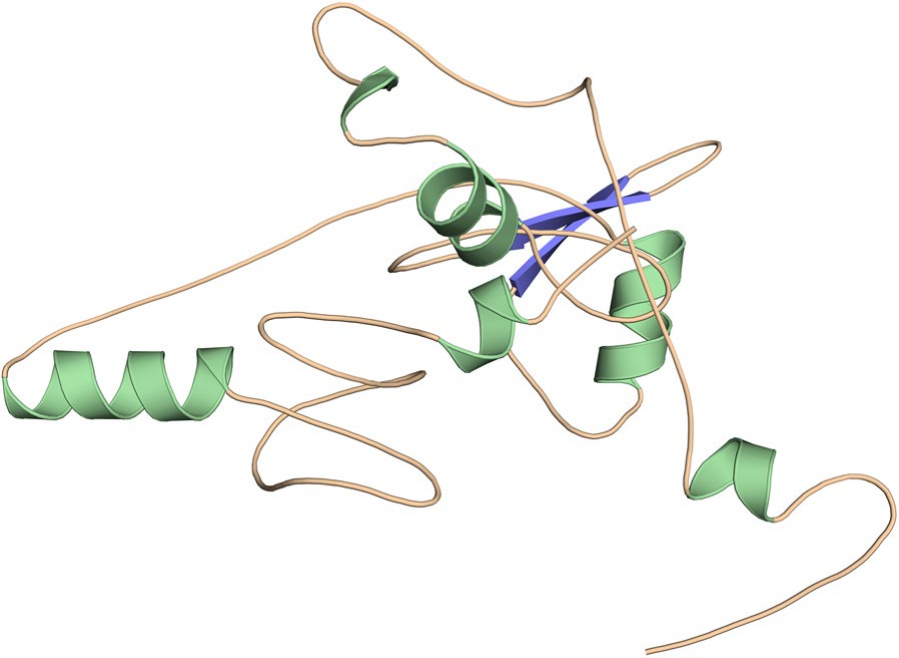
Homology modeling of the three-dimensional structure of the final multi-epitope vaccine construct

**Fig. 5:**
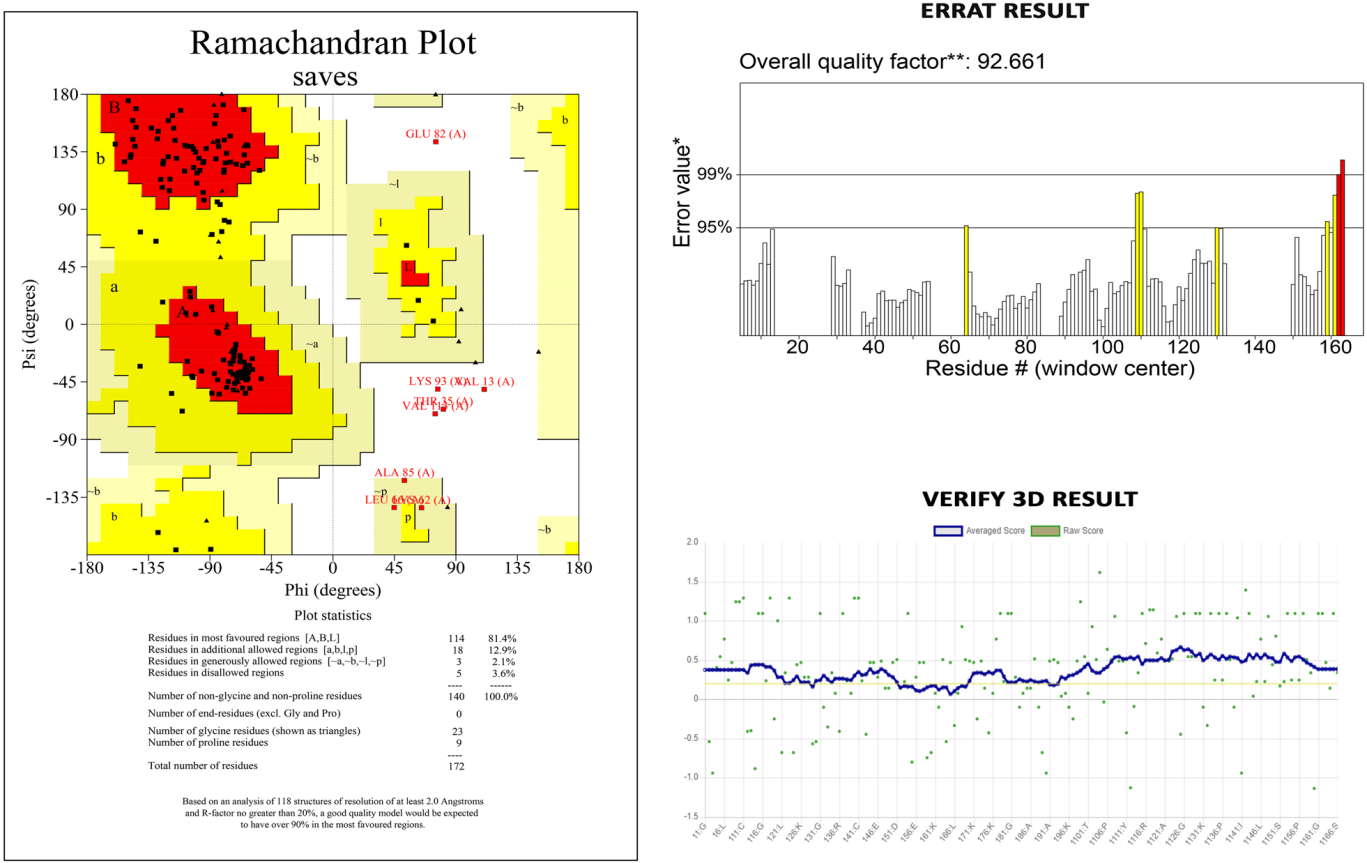
Several structure validations tools results confirmed the modeled multi-epitope vaccine structure to be reliable and accurate

The Robetta server was used to predict five tertiary structures for the vaccine construction, which were then analyzed using a Ramachandran plot to choose the optimum model. Model 3 was chosen because it provided the best arrangement of residues in allowed regions (Fig. 4). The Ramachandran plot showed that most of the residues were found in the favored (81.4%), allowed (12.9%) regions, and 2.1% in generously allowed regions, while 3.6% residue was present in the disallowed region. The model's ERRAT quality factor and Verify-3D score were 92.661 and 95.93; respectively indicate that this structure is energetically stable. The Ramachandran plot, ERRAT, and verify3D of the multi-epitope peptide structure are shown in Fig. 5.

### Disulfide engineering for vaccine protein stability

**Fig. 6:**
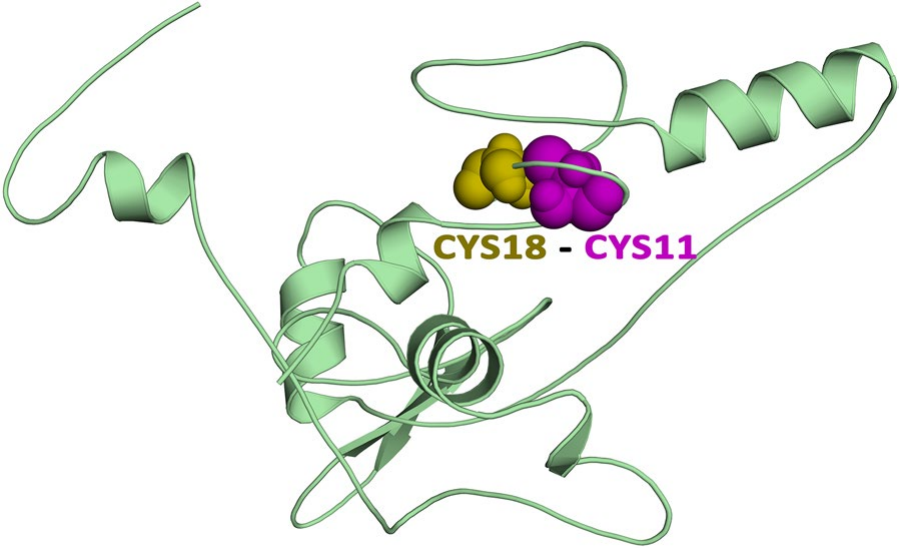
Disulphide engineering of the vaccine protein. Residue pairs showed in purple (CYS11) and olive (CYS18) spheres were mutated to Cysteine residues to form disulphide bridge between them

After running the vaccine sequence through the Disulfide by Design 2.13 tool, a total of 13 possible residue pairings that potentially form a disulfide bond were found, as shown in Additional file 6: Table S6. Taking the bond energy and χ3 parameters into consideration, only one pair of residues was chosen because their scores fit conventional standards, i.e. the bond energy should be less than 2.2 kcal/mol and the χ3 angle should be between −87° and + 97°. As illustrated in Fig. 6, a mutation pair was created on the residue pair CYS11–CYS18, which had bond energy of 1.14 kcal/mol and a χ3 angle of + 96.08°.

### Mapping of discontinuous B-cell epitopes in the vaccine protein

**Fig. 7:**
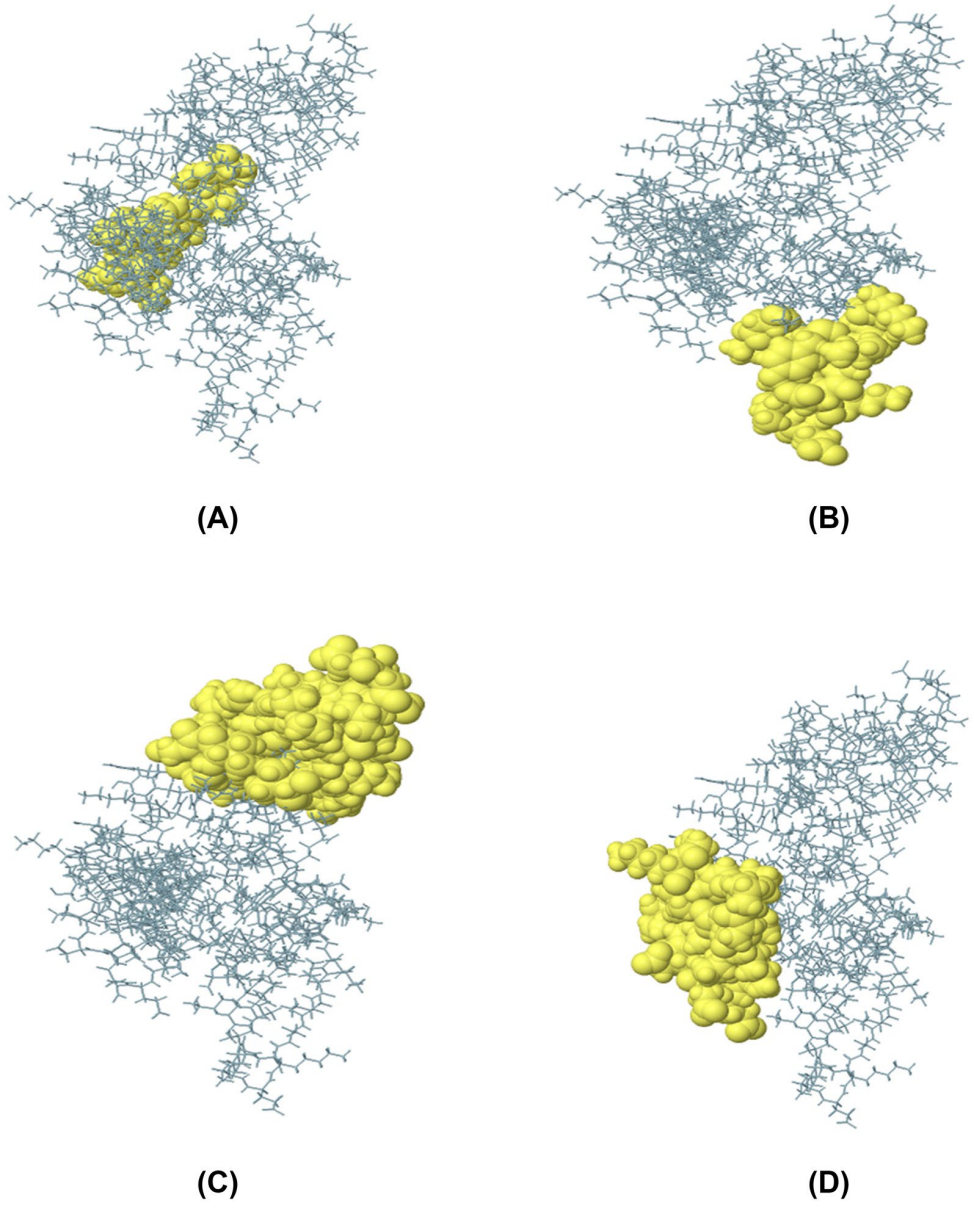
The conformational B-lymphocyte epitopes present in the vaccine. The yellow spheres showing epitopes containing (**A**) 20 residues (AA 153–172) with 0.809; (**B**) 14 residues (AA 69, AA 71, and AA 88–99) with 0.745; (**C**) 39 residues (AA 11, AA 13–16, AA 24–40, AA 42, and AA 44–59) with 0.621; (**D**) 27 residues (AA 1–2, and AA 127–151) with 0.596

B lymphocytes boost humoral immunity by secreting antibodies and cytokines that neutralize foreign antigens [38, 39] That's why it is essential to have a sufficient number of B-cell epitopes inside the protein domain. The existence of conformational B-cell epitopes was analyzed using the ElliPro tool of the IEBD server, which found 4 conformational B-cell epitopes of 14–39 residues (Additional file 7: Table S7) with scores ranging from 0.596–0.809, as shown in Fig. 7.

### Molecular docking of vaccine with TLR4 receptor

**Fig. 8:**
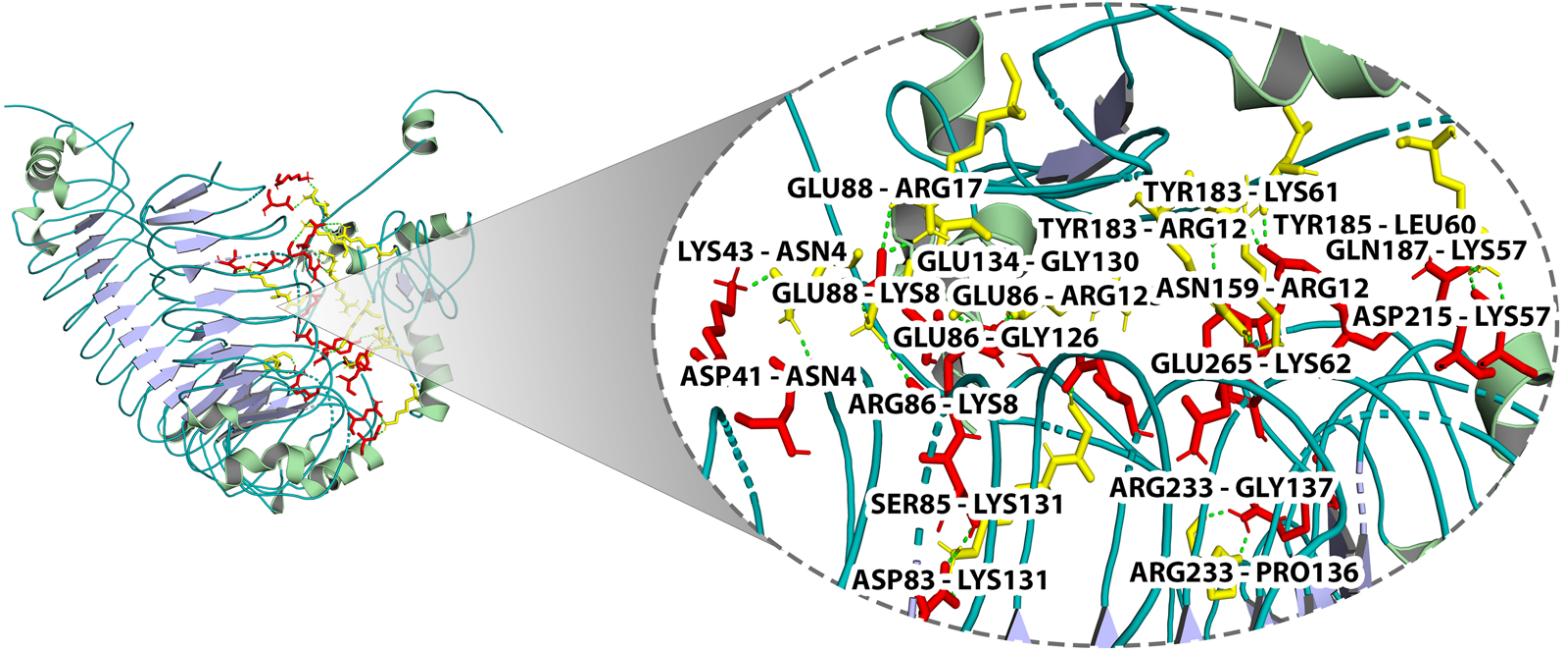
Molecular interaction of multi-epitope vaccine construct docked with TLR2

To assess the binding affinity between the vaccine construct and TLR4, docking analysis of vaccine protein with TLR4 was undertaken. Molecular docking was performed by using the grid box set in the active site region. The lowest binding energy was –856.8, and the center energy between the ligand and receptor was −671.1. Pymol was used to visualize the interactions between the TLR4 receptor and the vaccine candidate, and the docked complex is shown in Fig. 8. Residues of the vaccine construct and TLR4 receptor were found to make polar contacts are LYS43-ASN4, ASP41-ASN4, GLU88-ARG17, GLU88-LYS8, ARG86-LYS8, SER85-LYS131, ASP83-LYS131, GLU86-GLY126, GLU86-ARG125, GLU134-GLY130, ARG233-GLY130, ARG233-GLY130, ARG233-PRO136, GLU265-LYS62, TYR183-LYS61, TYR183-ARG12, ASN159-ARG12, TYR185-LEU60, GLN187-LYS57, and ASP215-LYS57 with the distance of 1.7 Å, 1.9 Å, 1.8 Å, 1.8 Å, 2.4 Å, 1.8 Å, 1.7 Å, 1.8 Å, 1.7 Å, 2.3 Å, 1.7 Å, 1.8 Å, 1.7 Å, 2.2 Å, 1.8 Å, 1.7 Å, 1.6 Å, 1.9 Å, 1.7 Å and 1.8 Å, respectively.

### Molecular dynamic (MD) simulation

**Fig. 9:**
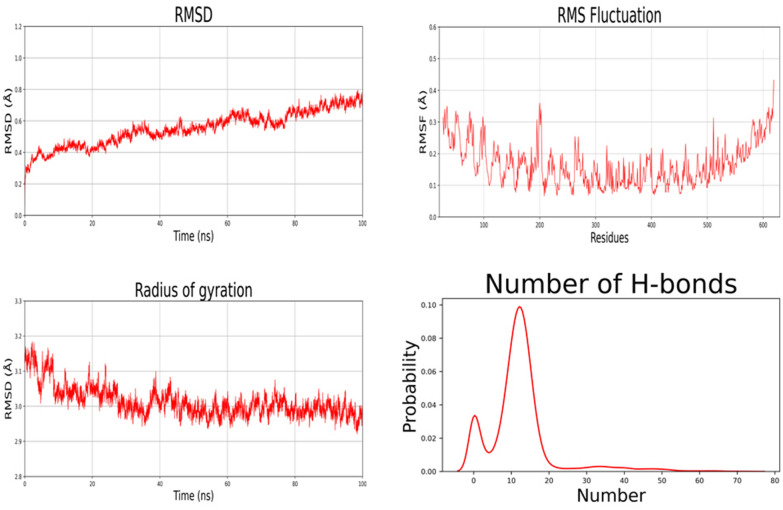
Root mean square deviation (RMSD) and root mean square fluctuation (RMSF) analysis of protein backbone and side chain residues of MD simulated vaccine construct

MD simulation in an aqueous environment was used to assess the stability of the protein–protein complex. The simulation time was displayed against the produced vaccine-TLR4 complex. according to the results, the cubic box was successfully created under intermittent boundary circumstances, and neutralization and energy reduction were carried out efficiently. Here we have run a simulation for 100nsffa. The produced vaccine's RMSD plot (as a ligand) indicated values in the 0.1–0.2 nm range. Result of the root mean square deviation has been shown in Fig. 9. It is noteworthy that the system was equilibrated in a shorter period. The RMSF plot shows that the complex is overlapped except at the N and C terminal of the protein as the substrate is interacting in this region (Fig. 9). The Substrate interacting region of the system is more fluctuating.

The hydrogen bond is vital in preserving protein secondary structure, therefore an accurate description of hydrogen bond interaction is critical in protein folding simulation. Hydrogen bonding is an important nonbonded interaction in system simulation, which is dominated by electrostatic interaction. The number of hydrogen bonds is calculated using a sigmoidal function of the donor–acceptor distance as a function of hydrogen bond strength. The peak in Fig. 9 is quite high, demonstrating that integrating the polarisation effect strengthens hydrogen bonding. Furthermore, any modification in protein motif and shape can be evaluated by Gyration Radius (Fig. 9). The spherical state information about total protein volume distribution is obtained by this parameter. The results suggest that the Gyration Radius of the complex is more downwards that is a clue of constructed secondary structures and it seems that this is a result of the limitation of the main chain movement of the system.

### *Codon adaptation and *in silico* cloning*

**Fig. 10:**
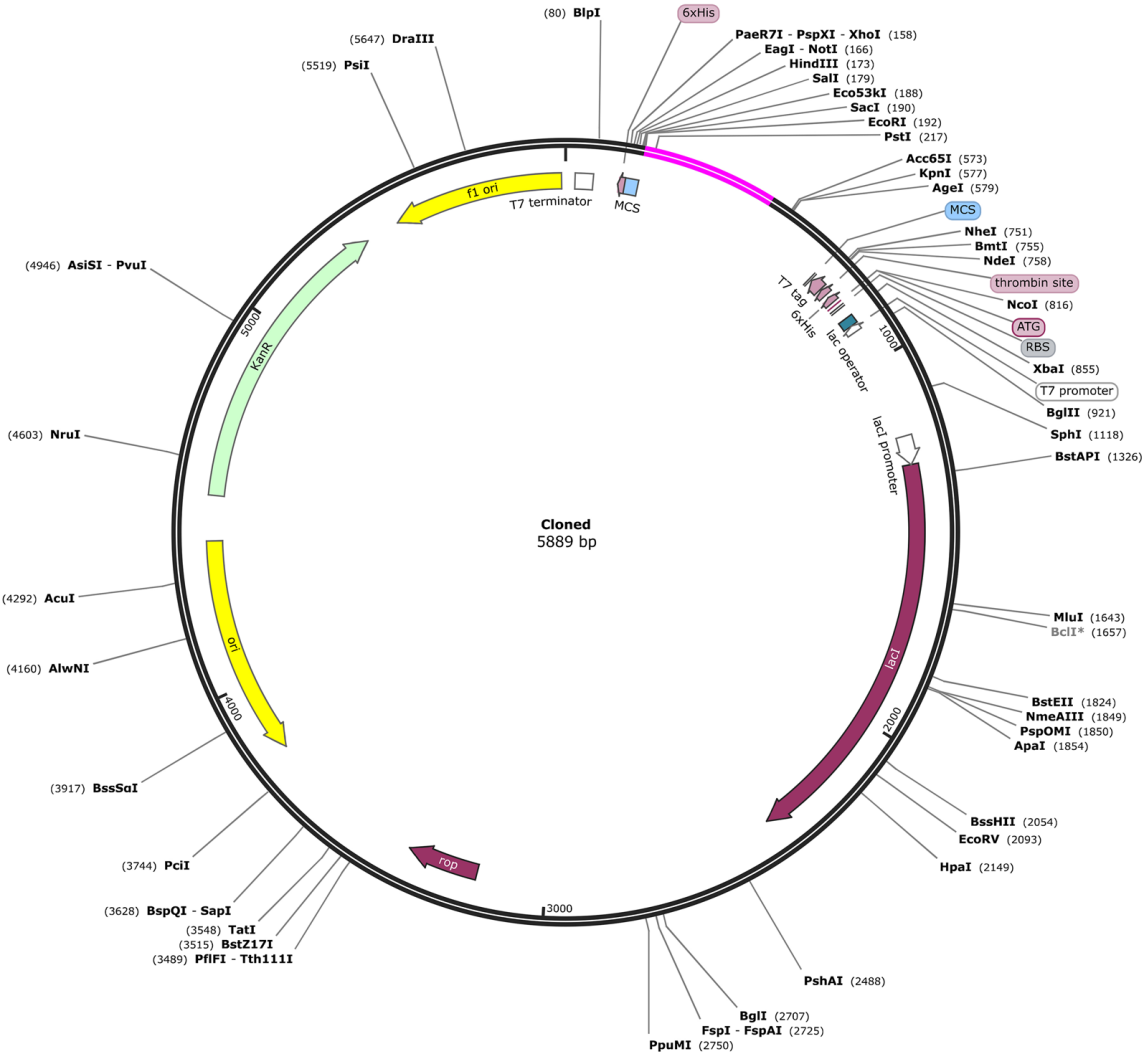
Restriction cloning of final multi-epitope vaccine by using pET28a (+) expression vector in the in silico space. Black circle indicates the vector, and the magenta part is the place where the vaccine is inserted

Since differences in codon use result in limited translation of foreign genes, codon adaptation is the greatest strategy to improve translational efficiency. The JCat tool was utilized to improve our developed vaccine's codon use in relation to the *E. coli* K12 strain. The optimized sequence had a GC content of 51.93%, indicating effective expression in the *E. coli* host with a Codon adaptation index (CAI) of 1. The results showed that following adaptation, the prokaryotic ribosome binding site, restriction enzyme cleavable sites and rho independent transcription terminators were all eliminated. Following that, the vaccine construct's modified codon sequence was introduced into the *E. coli* expression vector PET28a (+) between the EcoRI and BamHI restriction sites, as shown in Fig. 10. A 6-histidine tag was also inserted to facilitate immune-chromatographic purification of the recombinant vaccine. As a result, the clone's length was 5889 bp.

## In silico immune response simulation

**Fig. 11:**
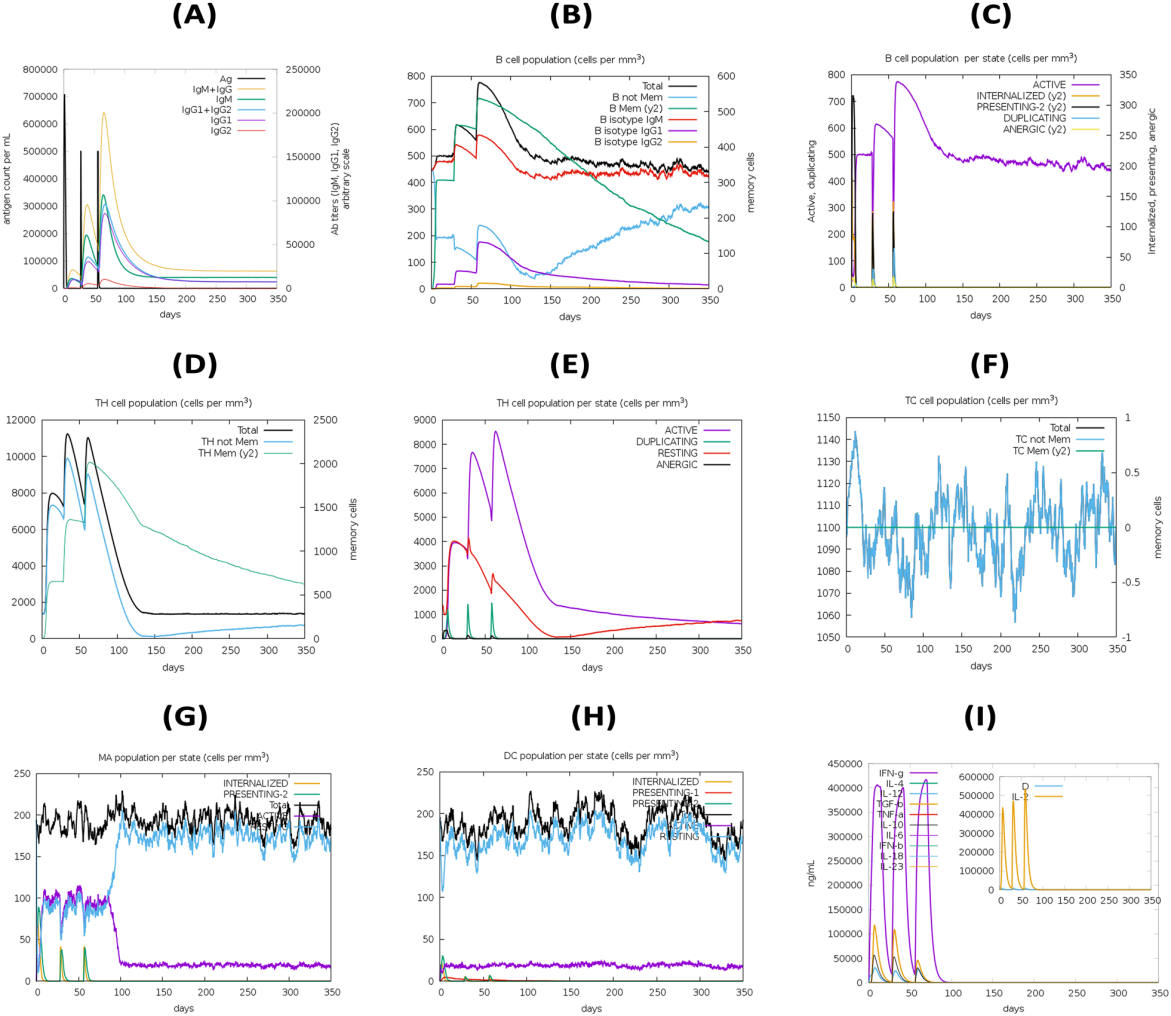
In silico simulation of immune response using vaccine as antigen: **A** antigen and immunoglobulins, **B** B-cell population, **C** B-cell population per state, **D** helper Tcell population, **E** helper T-cell population per state, **F** cytotoxic T-cell population per state, **G** macrophage population per state, **H** dendritic cell population per state, and **I** production of cytokine and interleukins with Simpson index D of immune response

The C-ImmSim results show that the main immune response following vaccine administration is considerably promoted by the steady increase in immunoglobulin levels (i.e., IgM, IgG1, IgG2, IgG1 + IgG2, and IgG + IgM) during the secondary and tertiary immunological responses (Fig. 11A). During the secondary and tertiary immune responses, the antigen level fell as well. Furthermore, the raised and maintained concentrations of the B-cell population (total, active, and plasma B-cell) and T-cell (helper T-cell, cytotoxic T-cell, regulatory T-cell) (Fig. 11B–F) clearly indicated the efficacy of the designed vaccine. After each dose, the concentration of Th1 was shown to rise. Furthermore, a higher density of dendritic and macrophage cells suggests that antigen-presenting cells efficiently process and deliver antigen to CD4+  and CD8 + cells (Fig. 11G-H). It is also obvious that the levels of several cytokines rose following exposure (Fig. 11I).

## Discussion

Vaccination is a widely employed technique for improving the host immune mechanism against a specific pathogen [40, 41]. Producing a range of vaccines, such as live or attenuated vaccines, is expensive, time-consuming, and takes a long time to reach the market [42, 43]. Moreover, the attenuated vaccine delivers poor passive immunization and causes allergic responses due to the large antigenic load [44]. With the advancement of multi-omics technology, it is now easier for identification of epitopes that elicit strong immune response. Multi-epitopes-driven vaccines against *N.* *meningitidis* and *M. leprae* have been developed utilizing immunoinformatics methodologies [45, 46].

*Enterococcus faecium* is a life-threatening bacterium that has recently arisen at an alarming pace. Limited advances have been made in the elucidation of the host immune response against invasive *E. faecium* infections. The innate immune defense system depends on the recognition of the pathogen-associated molecular patterns (PAMPs). PAMPs are recognized through pattern recognition receptors, e.g., the components of the complement system and the Toll-like receptors (TLRs). There is evidence that TLR 2 (Toll-like receptor 2) plays a significant role in the innate immune response against *E. faecium* by recognizing peptidoglycan and lipoteichoic acid. Apart from this direct interaction of the pathogen with the phagocyte, there is also an indirect pathway mediated through opsonins, comprised of immunoglobulins and complement components. Activation of the alternative complement pathway elicits deposition of the complement component on the bacterial surface, which is subsequently recognized by complement receptors on the phagocytes [47]. The incompetence of the immune system to kill the intracellular enterococci may lead to their systemic spread.

As a result, developing possible vaccinations might be a boon in the battle against this bacteria. A possible vaccine has been designed as an optimal option to prevent *E. faecium* infection in the current investigation. Penicillin-binding protein, a protein associated with the bacteria's pathogenicity, was chosen based on its antigenicity score, since the higher the antigenicity, the greater the immune response. Various databases were used to anticipate potential T and B-cell epitopes, and their viability for use as a vaccine candidate was investigated. CTL epitopes help in the recognition of foreign antigen fragments on MHC-I (major histocompatibility complex-I) molecules and destroy target cells whereas HTL epitopes are required for both humoral and cell-mediated immune responses. B-cell epitopes are also significant in the development of epitopic vaccines as well as disease detection**.** As a result, prediction of these epitopes are critical for the creation of an immunotherapeutic and preventive vaccination. Antigenicity prediction of the protein sequence is important because it indicates whether the bacterial protein sequence is likely to be recognized by immunogenic cells in the human body. ABCpred, NetMHCpan 4.1, and IEDB MHC II prediction tools was used to predict putative B-cell, CTL, and HTL epitopes. All of the epitopes were found to be ‘antigenic’ as analyzed by the VaxigenV2.0 server. Another prominent obstacle in vaccine development is the probability of allergenicity since many vaccines stimulate the immune system into an allergenic reaction. AllerTOP server was used to predict potential allergenicity and all of the epitopes were observed as ‘non-allergen". IEDB class I immunogenicity tool was utilized to find the immunogenicity score of the peptides. Toxinpred server identified all peptides as ‘non-toxin’ along with hydrophobicity, hydropathicity, hydrophilicity, charge, SVM score, and molecular weight of the predicted peptides. Finally, two LBL, five CTL, and two HTL epitopes were chosen based on their conservancy, interaction with the greatest number of HLA alleles, antigenicity, immunogenicity, non-allergenicity, non-toxicity, and capacity to induce cytokine production. Geographical differences impact the development of subunit vaccines because they influence the variety of HLA allele expression throughout the world [48, 49]. The global population coverage of the finalized T-cell epitopes (CTL and HTL) with their corresponding HLA alleles was observed to be 99.25 %. Finally, to ensure an immune response, KK, AAY, and GPGPG linkers were inserted between the LBL, CTL, and HTL epitopes to create a stable and effective vaccine design. β-defensins, a TLR4 agonist, was employed as an adjuvant and coupled to the N-terminal of the vaccine design using an EAAAK linker to increase the immunological response.

The final developed vaccine is 172 amino acids long and has a molecular weight of 18,294.07 Da, which falls within the optimal molecular weight range for chimeric vaccines. The suggested vaccine's pI value (9.38), instability index (18.85), GRAVY score (−0.472), and aliphatic index (73.90) reflect its basic, stable, hydrophilic, and thermostable characteristics, respectively. Furthermore, the vaccine's antigenicity (1.1569) and non-allergenicity further demonstrated its efficacy as a vaccination. Because better solubility of the recombinant protein within the *E. coli* host leads to faster separation and purification, solubility is one of the most fundamental and critical factors in vaccine manufacture. When the vaccine protein was overexpressed in the *E. coli* host, it was discovered to be soluble. Short half-lives of epitopes are often a worry while constructing vaccines and the proposed chimeric vaccine successfully demonstrated an acceptable stability index, indicating its potential as a vaccine candidate.

The secondary structure of the intended protein was expected to have 15.11 % helix, 27.32 % strands, and 57.55 % random coils after conformational analysis. Ramachandra plot showed that final vaccine protein have 81.4 % amino acid in the favorable section, 12.9 % amino acid in the additionally allowed zone, and 3.6 % amino acid in the prohibited region, indicating that the designed model is satisfactory. Furthermore, the ERRAT's Z-score (92.661) and Verify3D showed that the structural quality of the vaccine construct was promising. As a consequence of the validation results, the tertiary structure appears to be suitable for future investigation.

Increasing protein stability is conferred the utmost importance in various biomedical and therapeutic applications. Disulfide engineering and a pair of mutations on the CYS11-CYS18 residue pair were used to improve the protein's thermostability*.* The *E.coli* cell culture system is commonly utilized for bulk recombinant protein synthesis. As a result, codon optimization for *E. coli* strain K12 was carried out in order to achieve effective expression in the host. To ensure a higher degree of protein expression in *E. coli*, a GC content of 51.93 % was found, along with a codon adaptability index (CAI) of 1. Furthermore, the molecular docking experiment demonstrated that the proposed vaccine binds with low binding energy (856.6 kcal/mol) at the TLR4 receptor-binding site, demonstrating higher binding affinity. The stability and mobility of the TLR4-vaccine complex in the biological environment were assessed using a molecular dynamics simulation of the docked complex. The graph also showed that the protein–protein combination is extremely stable, with each residue exhibiting a decreased degree of deformation. Overall, molecular dynamics studies revealed that the suggested vaccine is sufficiently stable and has a low risk of deformability at the molecular level.

In addition, the immunological simulation results demonstrated that the vaccine design elicited an immune cascade against *E. faecium* that closely resembled the normal immune response to viral infections. Secondary and tertiary immune responses were shown to be stronger than primary immune responses after vaccination, with large amounts of antibodies produced and antigen cleared. The vaccination boosted both cell-mediated and humoral immunity, as seen by a rise in B-cells (memory B-cell and plasma B-cell) and T-cells (cytotoxic and helper T-cell). The vaccine design is capable of eliciting adequate antigen processing and presentation to CD4+ and CD8+ cells, as evidenced by the increased concentration of antigen processing cells such as dendritic cells and macrophages. However, further in vitro and in vivo experimental testing is needed to corroborate the in silico findings.

## Conclusion

The present work offers a novel multi-epitope vaccine construct against *E. faecium*. Combiniation immunoinformatics analysis, protein structure assessment, physicochemical and molecular dynamics studies confirmed the proposed vaccine to exibit strong humoral and cellular immune response and holds a strong reasons for further experimental validation.

## Supplementary Information

**Additional file 1: Table S1.** Predicted B-cell epitopes epitopes from *Enterococcus faecium* penicillin binding protein and their corresponding immunogenic properties. The final selected epitopes to design multi-epitope vaccine construct from proteins has been highlighted in green colour.

**Additional file 2: Table S2.** Predicted CTL epitopes from *Enterococcus faecium* penicillin binding protein with their corresponding MHC Class I alleles and their immunogenic properties. The final selected epitopes to design multi-epitope vaccine construct from proteins has been highlighted in green colour.

**Additional file 3: Table S3.** Predicted HTL epitopes from *Enterococcus faecium* penicillin binding protein with their corresponding MHC Class II alleles and their immunogenic properties. The final selected epitopes to design multi-epitope vaccine construct from proteins has been highlighted in green colour.

**Additional file 4: Table S4.** Population coverage analysis of the final vaccine construct using the population coverage analysis tool of the IEDB database by keeping the default parameters on (109 countries covering 16 different geographical regions).

**Additional file 5: Table S5.** Physicochemical properties, secondary structure, and solubility analysis of final vaccine construct as predicted by ProtParam tool, PSIPRED, and SOLpro server.

**Additional file 6: Table S6.** Disulfide engineering of the final multi-epitope vaccine.

**Additional file 7: Table S7.** Discontinuous B-cell epitopes with their scores predicted by ElliPro.

## Data Availability

All data generated or analysed during this study are included in this published article (and its Additional files).
