# Supplementary material for: Designing a novel multi-epitope vaccine to evoke a robust immune response against pathogenic multidrug-resistant Enterococcus faecium bacterium
Source: Gut Pathog. 2022 May 27;14:21. doi: 10.1186/s13099-022-00495-z (PMC9137449; doi:10.1186/s13099-022-00495-z)
Supplement: Supplementary file 5 — Additional file 5: Table S5. Physicochemical properties, secondary structure, and solubility analysis of final vaccine construct as predicted by ProtParam tool, PSIPRED, and SOLpro server. [file 13099_2022_495_MOESM5_ESM.docx]

**Supplementary Table 5.** Physicochemical Properties, secondary structure, and solubility analysis of final vaccine construct as predicted by ProtParam tool, PSIPRED, and SOLpro server.

| **Parameters** | **Vaccine Construction Sequence** |
| --- | --- |
| Number of amino acids | 172 |
| Molecular weight | 18294.07 Da |
| Theoretical pI | 9.38 |
| Total number of negatively charged residues (Asp + Glu) | 19 |
| Total number of positively charged residues (Arg + Lys) | 30 |
| Instability index | 18.85 (Stable) |
| Aliphatic index | 73.90 |
| Grand average of hydropathicity (GRAVY) | -0.472 |
| The estimated half-life | 30 hours (mammalian reticulocytes, in vitro).  >20 hours (yeast, in vivo)  >10 hours (Escherichia coli, in vivo). |
| Extinction coefficients | 30285 |
| Amino acid composition | \| Ala (A) \| 20 \| 11.6% \| \| --- \| --- \| --- \| \| Arg (R) \| 9 \| 5.2% \| \| Asn (N) \| 3 \| 1.7% \| \| Asp (D) \| 11 \| 6.4% \| \| Cys (C) \| 6 \| 3.5% \| \| Gln (Q) \| 3 \| 1.7% \| \| Glu (E) \| 8 \| 4.7% \| \| Gly (G) \| 23 \| 13.4% \| \| His (H) \| 0 \| 0.0% \| \| Ile (I) \| 9 \| 5.2% \| \| Leu (L) \| 14 \| 8.1% \| \| Lys (K) \| 21 \| 12.2% \| \| Met (M) \| 1 \| 0.6% \| \| Phe (F) \| 2 \| 1.2% \| \| Pro (P) \| 9 \| 5.2% \| \| Ser (S) \| 5 \| 2.9% \| \| Thr (T) \| 12 \| 7.0% \| \| Trp (W) \| 1 \| 0.6% \| \| Tyr (Y) \| 9 \| 5.2% \| \| Val (V) \| 6 \| 3.5% \| \| Pyl (O) \| 0 \| 0.0% \| \| Sec (U) \| 0 \| 0.0% \| |
| Atomic composition | \| Carbon \| C \| 806 \| \| --- \| --- \| --- \| \| Hydrogen \| H \| 1311 \| \| Nitrogen \| N \| 227 \| \| Oxygen \| O \| 243 \| \| Sulfur \| S \| 7 \| |
| Secondary structure analysis | Alpha helix (Hh) XX.XX%  Extended strand (Ee) XX.XX%  Random coil (Cc) XX.XX% |
| Solubility (SOLpro Server) | SOLUBLE with probability 0.500000 |
