# Supplementary material for: Designing a novel multi-epitope vaccine to evoke a robust immune response against pathogenic multidrug-resistant Enterococcus faecium bacterium
Source: Gut Pathog. 2022 May 27;14:21. doi: 10.1186/s13099-022-00495-z (PMC9137449; doi:10.1186/s13099-022-00495-z)
Supplement: Supplementary file 7 — Additional file 7: Table S7. Discontinuous B-cell epitopes with their scores predicted by ElliPro. [file 13099_2022_495_MOESM7_ESM.docx]

**Supplementary Table 7:** Discontinuous B-cell epitopes with their scores predicted by ElliPro.

| **Sl. No.** | **Residue** | **Number of Residue** | **Score** |
| --- | --- | --- | --- |
| A | A:G153, A:P154, A:G155, A:P156, A:G157, A:D158, A:S159, A:L160, A:G161, A:G162, A:K163, A:A164, A:G165, A:S166, A:T167, A:V168, A:A169, A:T170, A:T171, A:P172 | 20 | 0.809 |
| B | A:D69, A:K71, A:K88, A:L89, A:I90, A:A91, A:D92, A:K93, A:E94, A:T95, A:K96, A:A97, A:A98, A:Y99 | 14 | 0.745 |
| C | A:C11, A:V13, A:R14, A:G15, A:G16, A:L24, A:P25, A:K26, A:E27, A:E28, A:Q29, A:I30, A:G31, A:K32, A:C33, A:S34, A:T35, A:R36, A:G37, A:R38, A:K39, A:C40, A:R42, A:K44, A:K45, A:E46, A:A47, A:A48, A:A49, A:K50, A:D51, A:A52, A:D53, A:G54, A:V55, A:E56, A:K57, A:K58, A:V59 | 39 | 0.621 |
| D | A:G1, A:I2, A:T127, A:T128, A:G129, A:G130, A:K131, A:L132, A:G133, A:P134, A:G135, A:P136, A:G137, A:D138, A:K139, A:F140, A:I141, A:F142, A:G143, A:E144, A:D145, A:L146, A:D147, A:L148, A:P149, A:I150, A:S151 | 27 | 0.596 |
